# Evaluation of the Diagnostic Potential of Recombinant *Coxiella burnetii* Com1 in an ELISA for the Diagnosis of Q Fever in Sheep, Goats and Cattle

**DOI:** 10.3390/microorganisms8081235

**Published:** 2020-08-13

**Authors:** Mareike Stellfeld, Claudia Gerlach, Ina-Gabriele Richter, Peter Miethe, Dominika Fahlbusch, Birgitta Polley, Reinhard Sting, Martin Pfeffer, Heinrich Neubauer, Katja Mertens-Scholz

**Affiliations:** 1 Friedrich-Loeffler-Institut, Federal Research Institute for Animal Health, Institute of Bacterial Infections and Zoonoses, 07743 Jena, Germany; mareike.stellfeld@fli.de (M.S.); claudia.gerlach@gmx.de (C.G.); heinrich.neubauer@fli.de (H.N.); 2 Research Centre of Medical Technology and Biotechnology (Forschungszentrum für Medizintechnik und Biotechnologie GmbH), 99947 Bad Langensalza, Germany; irichter@fzmb.de (I.-G.R.); pmiethe@fzmb.de (P.M.); dfahlbusch@fzmb.de (D.F.); 3 Chemical and Veterinary and Investigations Office (Chemisches und Veterinäruntersuchungsamt), 70702 Fellbach, Germany; Birgitta.Polley@cuvas.bwl.de (B.P.); Reinhard.Sting@cuvas.bwl.de (R.S.); 4 Institute of Animal Hygiene and Veterinary Public Health, University of Leipzig, 04103 Leipzig, Germany; pfeffer@vetmed.uni-leipzig.de

**Keywords:** *C. burnetii*, Q fever, Com1, antigen, ELISA, diagnostic, serology

## Abstract

*Coxiella burnetii* is the causative agent of Q fever, a zoonosis infecting domestic ruminants and humans. Currently used routine diagnostic tools offer limited sensitivity and specificity and symptomless infected animals may be missed. Therefore, diagnostic tools of higher sensitivity and specificity must be developed. For this purpose, the *C. burnetii* outer membrane protein Com1 was cloned and expressed in *Escherichia coli*. The His-tagged recombinant protein was purified and used in an indirect enzyme-linked immunosorbent assay (ELISA). Assay performance was tested with more than 400 positive and negative sera from sheep, goats and cattle from 36 locations. Calculation of sensitivity and specificity was undertaken using receiver operating characteristic (ROC) curves. The sensitivities and specificities for sheep were 85% and 68% (optical density at 450nm, OD_450_ cut-off value 0.32), for goats 94% and 77% (OD_450_ cut-off value 0.23) and for cattle 71% and 70% (OD_450_ cut-off value 0.18), respectively. These results correspond to excellent, outstanding and acceptable discrimination of positive and negative sera. In summary, recombinant Com1 can provide a basis for more sensitive and specific diagnostic tools in veterinary medicine.

## 1. Introduction

*Coxiella burnetii*, the etiological agent of Q fever, is an intracellular, Gram-negative bacterium that causes local outbreaks in man and animals worldwide almost every year [1,2,3]. Domestic ruminants are considered as main reservoir hosts and infections in particular in sheep and goats are associated with human cases [4,5,6]. An infection in ruminants is often symptomless and serologically negative shedders have been reported [7]. Symptoms may vary from reproductive disorders, abortion, weak offspring to loss of performance finally causing economic losses [8]. As reproductive disorders can have different etiologies, Q fever is often not considered and the spread of *C. burnetii* via aerosols proceeds [9]. *C. burnetii* is highly contagious also for humans and is an occupational hazard for people in close contact with infected ruminants like farmers, shepherds and veterinarians as well as abattoir workers [5,6,10]. The typical course of disease is an acute, often self-limiting flu-like illness. Although the chronic form of human Q fever is less common, the consequences, e.g., endocarditis, encephalitis and hepatitis, are even more severe [11,12,13]. The small size of the bacterium, the high number of bacteria excreted via birth products, and aerosol transmission are the main reasons for large-scale outbreaks in populations living in close proximity to lambing mother herds as demonstrated during the Dutch outbreak between 2007 and 2010 [14]. More than 4000 acute and 284 chronic human cases were reported, mirrored by high numbers of seropositive blood donors [15,16].

Prevalences in animals depend on species, herd sizes, housing systems and geographical regions [2,17,18]. Seasonal differences and differences between years may occur [19], but reasons for these variations are poorly understood. Additionally, data also may vary due to the different serodiagnostic test systems used. In Germany, approximately 150 to 300 cases are notified in farm ruminants annually, but the prevalences and incidences vary on a regional scale [20]. In Thuringia, a federal state in Central Germany, seroprevalences were 10% in cattle and 28% in sheep, studies in Lower Saxony in Northern Germany showed seroprevalences of 2.7% in sheep, whereby migratory flocks of sheep showed intra-flock prevalences of up to 48% [18,21]. The prevalence of Q fever in farm animals is considerably high in almost every European country. In The Netherlands for example, seroprevalences of 82% in cattle and 31% to 79% in sheep flocks are reported [22,23]. In Denmark, 79% seropositive cattle herds were found [24]. A Polish study found a seroprevalence of 25% for dairy cattle herds [25]. In northwest Italy, antibodies against *C. burnetii* were detected in 39% and 20% of sheep and goat flocks, respectively [26]. Thus, the prevalence of Q fever varies greatly from one country to another.

Direct detection of *C. burnetii* can be achieved by polymerase chain reaction (PCR) or cultivation. The latter is difficult and time-consuming and is mostly applied when abortion material or milk is investigated for confirmation of a Q fever infection [27]. In veterinary medicine, indirect diagnostic methods such as enzyme-linked immunosorbent assays (ELISA) are used in routine diagnostics and for screening. For these commercially available ELISAs different sensitivities and specificities have been reported [28,29,30,31,32,33,34,35,36,37]. Thus, seronegative shedders may stay in the herds and circulation of *C. burnetii* continues. Commercial ELISAs used in veterinary diagnostics of Q fever are currently based on whole cell lysates of different *C. burnetii* strains. Furthermore, the specificity of tests can be influenced by cross-reactions to other pathogens [38,39,40,41,42]. While the IDEXX Q Fever Ab Test uses the *C. burnetii* Nine Mile phase I strain originally isolated from ticks, the IDvet ID Screen^®^ Q Fever Indirect Multi-species is based on a French bovine isolate and PrioCHECK™ Ruminant Q Fever Ab Plate Kit works with an ovine antigen phase I and II from an isolate from France [43,44,45]. Because whole cell antigens contain highly conserved proteins such as housekeeping proteins, the specificity can be influenced by cross-reactions with other pathogens such as *Chlamydia* spp. [40,46].

This problem could be solved by the use of a combination of *Coxiella*-specific proteins for highly sensitive and specific detection of *C. burnetii*-specific antibodies. The search for *C. burnetii*-specific serological markers has been intensified considerably in the last years [47]. In particular, chaperones such as the heat shock protein GroEL, the outer membrane chaperone OmpH and *Coxiella* outer membrane protein Com1, the peptidyl-prolyl cis-trans isomerase Mip as well as the surface protein YbgF were identified as potential *C. burnetii*-immunodominant proteins [48,49,50,51]. Xiong et al. demonstrated that recombinant proteins show a high reactivity with sera from patients with acute Q fever leading to a sensitivity of up to 88% in a protein microarray [48]. In that study, Com1 (*C. burnetii* unit (CBU) 1910) showed sensitivities in human sera with acute Q fever of 12%, with chronic Q fever of 52% and for convalescent patients of 50% and a specificity of 100%. Com1 as a chaperon catalyzes the formation of disulfide bonds in extra cytoplasmic proteins [52,53]. It has recently repeatedly been shown that Com1 as an antigen in an ELISA with human sera can lead to acceptable performance of the test with specificities of about 90% [54,55].

It is obvious that fast and reliable diagnosis of shedding animals is fundamental to any control program of *C. burnetii*. The aim of this study was to evaluate recombinant Com1 as ELISA antigen for use in Q fever diagnosis of ruminants. In the future, these data will allow the production of a pen side serodiagnostic test or other assay formats for pathogen detection.

## 2. Materials and Methods

### 2.1. Bacterial Strains, Plasmids and Culture Conditions

The experiments were carried out under biosafety level 2 conditions. Bacteria and plasmids used are listed in Table 1. *Escherichia coli* was grown at 37 °C in Luria–Bertani (LB) broth with constant gentle shaking at 180 rpm or on LB agar plates supplemented with spectinomycin (100 µg/mL) or ampicillin (100 µg/mL).

*Coxiella burnetii* Nine Mile phase II RSA 439 was grown in acidified citrate cysteine medium-2 (ACCM-2, Sunrise Science Products, Knoxville, TN, USA) at 37 °C with 2.5% O_2_ and 5% CO_2_. After 7 days of incubation, *C. burnetii* was harvested by centrifugation at maximum speed of 10,016× *g* for 20 min at 4 °C.

### 2.2. DNA Isolation

Genomic DNA of *C. burnetii* Nine Mile phase II strain RSA 439 was isolated with phenol–chloroform as described elsewhere [56]. Plasmid isolation from *E. coli* was carried out using the GenElute™ HP Plasmid Miniprep Kit (Sigma Aldrich, St. Louis, MO, USA) according to the manufacturers’ guidelines.

### 2.3. Cloning and Expression of Recombinant Coxiella burnetii Com1 Protein (CBU_1910)

**Figure A1:**
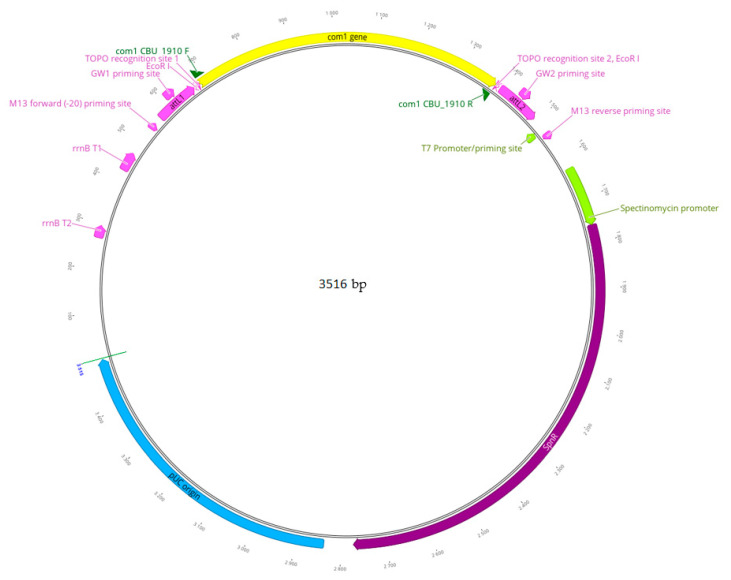
Vector map of recombinant pCR™8/GW/TOPO™ (Gateway, Invitrogen) with cloned *com1* (pCR8-*com1*).

**Figure A2:**
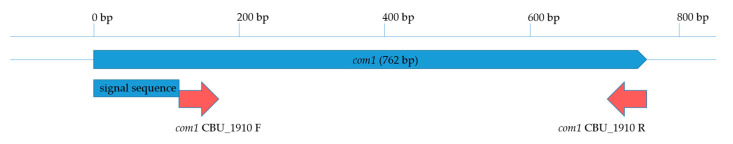
Cloned *com1*. Amplification of *com1* (762 bp) without the 5′-terminal signal sequence (1–60 bp) but including the 3′-terminal stop codon (759–762) from genomic DNA of *C. burnetii* Nine Mile Phase II RSA 439. Arrows show *com1* CBU_1910 F and *com1* CBU_1910 R primers used for amplification.

*Coxiella burnetii com1* (759 bp) was amplified without its signal sequence (1 to 63 bp) from genomic DNA of *C. burnetii* using *com1* CBU_1910 F primer (5′-ATGGCCCCCTCTCAATTCAGTT-3′) and *com1* CBU_1910 R primer (5′-CTACTTTTCTACCCGGTCGA-3′), respectively. The PCR reaction consisted of 0.5 µM of each primer, 4 µL 2× PCR Supermix (Invitrogen, Carlsbad, CA, USA) and 50 ng template DNA. An initial denaturation at 94 °C for 2 min was performed followed by 30 cycles of denaturation (94 °C for 15 s), annealing (53 °C for 30 s) and elongation (72 °C for 60 s) with a final elongation step at 72 °C for 10 min. The size of the PCR product was confirmed by agarose gel electrophoresis prior cloning into pCR™8/GW/TOPO™ (Gateway, Invitrogen, Carlsbad, CA, USA) as shown in Figure A1 (in Appendix A) and transformation of *E. coli* TOP10 (Invitrogen, Carlsbad, CA, USA) according to the manufacturer’s guidelines. Plasmids were isolated from overnight cultures, purified and sequenced (Eurofins Genomics, Ebersberg, Germany). Plasmids with correct insertion of *com1* were used for subcloning into pET300/NT-DEST (Gateway, Invitrogen, Carlsbad, CA, USA) as shown in Figure A2, named pET300-*com1* and sequenced for confirmation. All plasmids were stored at −20 °C.

Plasmid, pET300-*com1*, was transformed for expression into *E. coli* BL21(DE3) (Invitrogen, Carlsbad, CA, USA) according to the manufacturer’s protocol. Protein expression was induced during mid-exponentially growth with 0.4 mM isopropyl β-D-1-thiogalactopyranoside (IPTG) for two hours at 37 °C and constant gentle shaking at 180 rpm. Purification of the recombinant Com1 protein was carried out using nickel affinity chromatography according to the manufacturer’s guidelines of the ProBond™ Purification System (Invitrogen, Carlsbad, CA, USA) for native purification, with minor modifications. Briefly, bacteria (125 mL) were harvested by centrifugation (15 min, 3000× *g*, 4 °C) and lysed in 8 mL native buffer containing 8 mg lysozyme (Carl Roth, Karlsruhe, Germany). The lysate was subsequently loaded onto a 2 mL resin nickel-nitrilotriacetic acid (Ni-NTA, QIAGEN, Venlo, The Netherlands) column. To remove unbound proteins the column was washed twice with 8 mL native wash buffer containing 1.8 mM imidazole and twice with 8 mL native wash buffer containing 3.0 mM imidazole, respectively. Elution of recombinant Com1 protein was carried out by adding 6 mL elution buffer and six fractions of 1 mL each were collected. All fractions were analyzed by sodium dodecyl sulfate polyacrylamide gel electrophoresis (SDS-PAGE) followed by colloidal Coomassie Blue-staining and Western blot (see below). Fractions showing a band of approximately 29.9 kDa, the predicted molecular weight of recombinant Com1 protein including His-tag, were allied. The protein was concentrated using Vivaspin 10,000 MWCO (Sartorius, Göttingen, Germany) to a final volume of 1 mL. For protein concentration measurement, the Pierce™ 660 nm Protein Assay (Thermo Fisher, Waltham, MA, USA) was used according to the manufacturer’s guidelines using a Tecan Sunrise Remote Reader (Tecan Group Ltd., Zürich, Switzerland) with the software Magellan Standard V7.X. The purified recombinant Com1 protein was stored in elution buffer at −20 °C.

### 2.4. Sodium Dodecyl Sulfate Polyacrylamide Gel Electrophoresis (SDS-PAGE) and Western Blotting

His-tagged, purified recombinant Com1 protein was separated by SDS-PAGE on 4–20% Mini-PROTEAN^®^ TGX™ Precast polyacrylamide gel (BioRad, Hercules, CA, USA) according to the manufacturer’s protocol or using 12% Bis-Tris gels and SDS running buffer (25 mM Tris, 192 mM Glycine, 0.1% (*w*/*v*) SDS) as described elsewhere [56]. Proteins were stained with colloidal Coomassie Blue-staining (20% ethanol, 1.6% phosphoric acid, 8% ammonium sulfate, 1% Coomassie Brilliant Blue G250 (CBB G250; SERVA) in deionized water) overnight as given elsewhere and protein bands visualized by washing in deionized water [57]. For semi-dry immunoblotting separated proteins from polyacrylamide gels were immobilized on a polyvinylidene fluoride (PVDF) membrane (Merck, Darmstadt, Germany) in transfer buffer (48 mM Tris, 39 mM glycine, 1.3 mM SDS, 20% (*v*/*v*) methanol, pH 9.2) for 20 min at 20 V. For Western blot analysis, membrane was blocked with BlueBlock PF (SERVA, Heidelberg, Germany) for 1 h at room temperature and gently shaken. His-tagged recombinant Com1 protein was detected using anti-6xHis monoclonal mouse antibody (1:5000 in BlueBlock PF, Clontech) and traced with an alkaline phosphatase (AP) conjugated goat anti-mouse monoclonal antibody (1:5000 in BlueBlock PF, Sigma Aldrich, St. Louis, MO, USA) for 1 h at room temperature and shaken. Between each step, the membrane was washed three times in Tris-buffered saline-Tween (TBST; 150 mM NaCl, 20 mM Tris base, 0.05% Tween 20, *v*/*v*, pH 7.6) for 15 min at room temperature and gentle shaking. AP activity was detected with 0.33‰ (*w*/*v*) nitro blue tetrazolium (NBT, PanReac AppliChem, Darmstadt, Germany) and 0.17‰ (*w*/*v*) 5-bromo-4-chloro-3-indolyl-phosphate (BCIP, PanReac AppliChem, Darmstadt, Germany) in AP buffer (100 mM Tris base, 100 mM NaCl, 5 mM MgCl_2_, pH 9.5). Additionally, blotted SDS-PAGE-gels were stained with colloidal Coomassie blue for confirmation of the blot result.

### 2.5. Field Sera from Sheep, Goats and Cattle

Field sera from 111 sheep, 59 goats and 139 cattle were obtained from the National Reference Laboratory (NRL) for Q fever at the Friedrich–Loeffler Institute, Federal Research Institute for Animal Health (FLI). Among them were one commercially available Q fever negative lamb serum and 69 sheep sera from four locations, 6 goat sera from one location and 19 cattle sera from two locations tested directly and provided by the Chemical and Veterinary and Investigations Office Stuttgart (CVUA). A serum was considered Q fever true positive if gained from a herd or flock with animals showing clinical symptoms and following detection of *C. burnetii*-specific DNA or isolation of *C. burnetii* in the herd as well as with a serological positive result in a commercial ELISA for the serum (62 sheep sera from 4 different locations, 11 goat sera from two different locations, 43 cattle sera from 3 different locations). Additional sera positive for other pathogens were obtained from the respective National Reference Laboratory (NRL) of the FLI for brucellosis (3 goat and 2 cattle sera), chlamydiosis (4 sheep and 46 cattle sera) or paratuberculosis (42 goat and 10 cattle sera), respectively. Consequently 115 sheep, 104 goat and 197 cattle field sera were collected.

All 416 sera were tested for *C. burnetii*-specific antibodies using IDEXX Q Fever Ab Test (IDEXX), ID Screen^®^ Q Fever Indirect Multi-species (IDvet) ELISA or PrioCHECK Ruminant Q Fever Ab Plate Kit (Thermo Fisher Scientific, Waltham, MA, USA), respectively, according to the manufacturer’s guidelines. Results were evaluated according to the manufacturers’ guidelines. Briefly, using the IDEXX Q Fever Ab Test sera with an S/P% of ≥40% were considered positive, an S/P% between >30% and <40% suspicious or ≤30% negative. Using the ID Screen^®^ Q Fever Indirect Multi-species sera with an S/P% of ≥50% were considered positive, an optical density (OD) of >40% and <50% suspicious or ≤40% negative. Using the PrioCHECK Ruminant Q Fever Ab Plate Kit sera with an S/P% of ≥40% were considered positive or ≤40% negative. Of all 416 sera, 263 (100 sera of sheep, 49 sera of goats, 114 sera of cattle) were tested using a phase I/phase II antibody-specific ELISA (Euroimmun AG) according to the manufacturers’ guidelines. Briefly sera were considered positive with a ≥22 RU/mL (reactive units/mL), suspicious ≥16 to <22 RU/mL and negative with <16 RU/mL.

Sera tested are summarized in supplementary Table S1.

### 2.6. Indirect Enzyme-Linked Immunosorbent Assay (ELISA) Coated with Recombinant Com1 Protein (Com1-ELISA)

Recombinant Com1 protein was adjusted to 10 µg/mL in 50 mM Na_2_CO_3_, 50 mM NaHCO_3_, pH 9.6 and coated onto microtiter plates (Falcon, Corning, NY, USA; Sarstedt, Nümbrecht, Germany) by adding 100 µL protein solution per well. After an overnight incubation at 4 °C in a wet chamber, plates were blocked with 200 µL CPBS buffer per well (phosphate-buffered saline (PBS with 154 mM NaCl, 5.6 mM Na_2_HPO_4_, 1.1 mM KH_2_PO_4_, Lonza), 20% (*v*/*v*) Casein Buffer Concentrate (CBC1, Stereospecific Detection Technologies GmbH, Baesweiler, Germany) for 1 h at 37 °C in a wet chamber. The plates were washed three times with 300 µL/well of PBST (PBS, 0.05% Tween 20, *v*/*v*, pH 7.4) and 100 µL of field sera from sheep, goats or cattle were added in duplicate per well in a dilution of 1:64 in CPBS. Plates were incubated for 1 h at 37 °C in a wet chamber. After washing three times with 300 µL/well PBST, 100 µL/well of the secondary peroxidase-conjugated, monoclonal peroxidase conjugated Fc-specific anti-goat/sheep or peroxidase conjugated Fc-specific anti-bovine IgG antibody (1:50,000 in CPBS, Sigma), respectively, were added and incubated for 1 h at 37 °C in a wet chamber. After additional three washing steps with 300 µL/well PBST, 100 µL/well substrate solution (SeramunBlau^®^ slow2, Seramun Diagnostica GmbH, Heidesee, Germany) was added and incubated in the dark for 15 min at room temperature. The reaction was stopped by adding 100 µL/well of 1.25 M H_2_SO_4_ and OD at 450 nm (OD_450_) was measured (Tecan Sunrise Remote, Tecan Group Ltd., Zürich, Switzerland).

### 2.7. Statistical Analyses

All analyses were performed with Microsoft Excel Professional Plus 2016. OD_450_ values from Com1-ELISA were corrected by deletion of the highest result for all predicted negative sera and lowest result for all predicted positive sera. Data were categorized as positive or negative and arranged according to their OD_450_ values. Using a pivot table, sensitivity p(Com1 +|commercial ELISA +) and false-positive rate p(Com1 +|commercial ELISA-) as 1–specificity were calculated. Thereby, a receiver operating characteristic curve (ROC curve) was designed and the integral as the area under the curve (AUC) was calculated by trapezoidal method [58]. The cut-off values were defined at the OD_450_ where sensitivity and specificity were found to be highest using the pivot table and ROC curve as well as contingency table [59].

## 3. Results

### 3.1. Cloning and Expression of Com1

**Figure A3:**
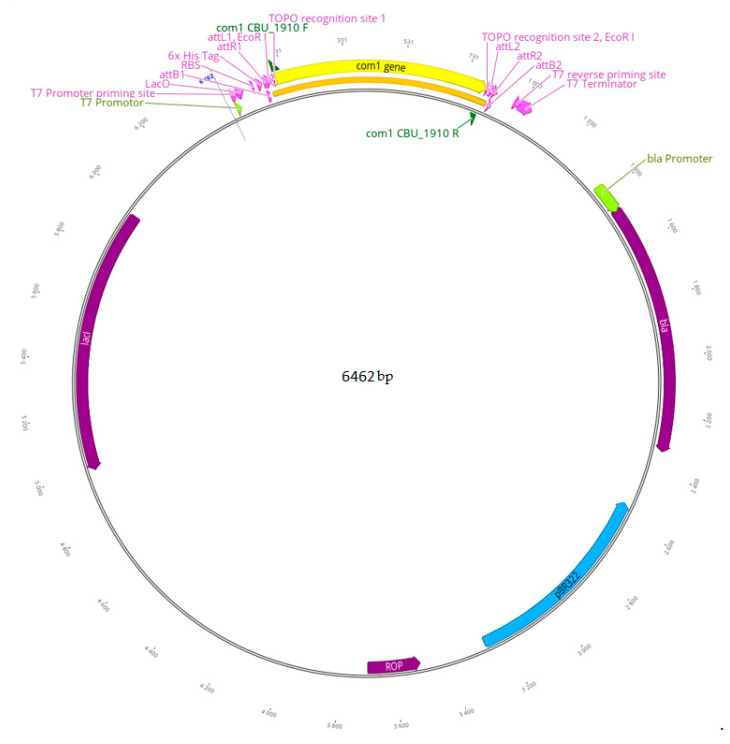
Vector map of recombinant expression vector pET300/NT-DEST (Invitrogen) with cloned *com1* cloned into (pET300-*com1*).

**Figure A4:**
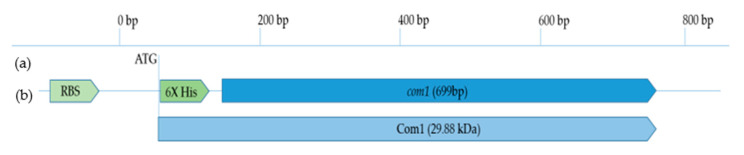
Cloned and expressed Com1 protein. (**a**) Insertion of *com1* into pET300/NT-DEST vector for expression of recombinant Com1 of 29.9 kDa with His-tag. (**b**) Expression of His-tagged *com1* as recombinant Com1 protein. ATG, vector encoded start codon; RBS, vector encoded ribosome binding site; 6xHis, vector encoded His-tag. Axis counting base pairs in relation to *com1* Figure A2.

*C. burnetii* Com1 (CBU_1910) is an outer membrane-associated protein (27.63 kDa) with several predicted membrane integration regions [52]. It consists of a secretory signal peptide (SignalP-5.0 Server, http://www.cbs.dtu.dk/services/SignalP/, DTU Health Tech, Lyngby, Denmark) with a predicted cleavage site between amino acid 21 and 22. The gene (762 bp) was amplified without its signal sequence (1–60 bp) but including its stop codon (759–762) for cloning into pET300/NT-DEST via pCR™8/GW/TOPO™ using the gateway system (Figure A1 and Figure A2). The final product pET300-*com1* was confirmed by sequencing and contained the truncated *orf com1* including fusion to a 5′-terminal His-tag for detection. The recombinantly expressed His-tagged protein Com1 had a predicted molecular weight of 29.88 kDa (Figure A3 and Figure A4). 

**Figure 1:**
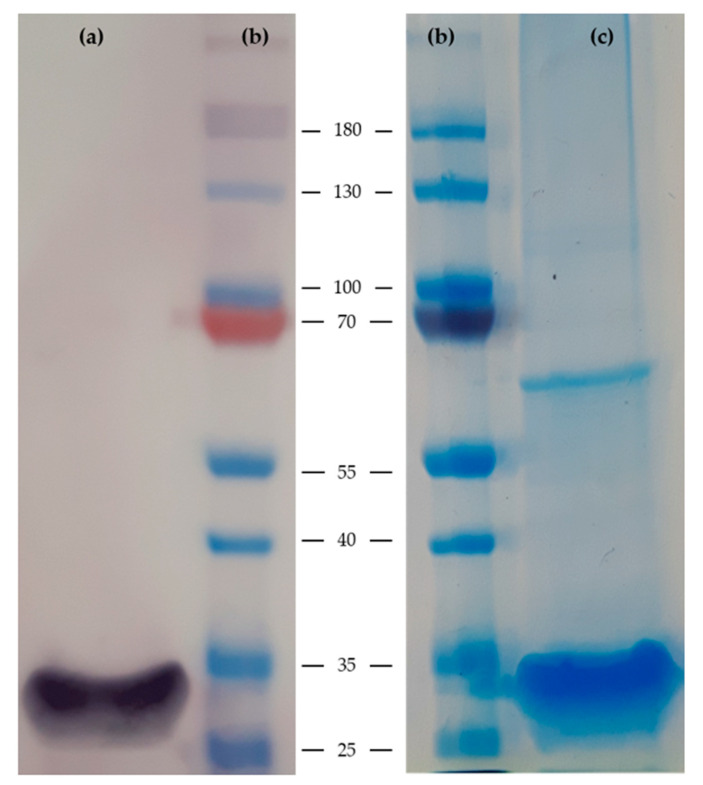
Analysis of purified recombinant Com1 protein with His-tag. Ni-NTA purified recombinant Com1 protein (predicted molecular weight 29.9 kDa, 30 µg per lane) was separated using a 12% sodium dodecyl sulfate polyacrylamide gel electrophoresis (SDS-PAGE). (**a**) For Western blot analysis mouse anti-6xHis monoclonal antibody (1:5000, Clontech Laboratories, Inc., Mountain View, CA, USA) was used and traced with alkaline phosphatase labeled goat anti-mouse secondary antibody (1:5000, Sigma-Aldrich, St. Louis, MO, USA). (**c**) Recombinant Com1 protein was visualized using colloidal Coomassie Blue. Lane (**a**,**c**), purified recombinant Com1 protein; Lane (**b**), protein marker with size in kDa (Thermo Fisher Scientific, Waltham, MA, USA).

Successful expression and purification of recombinant Com1 protein was confirmed by SDS-PAGE and Western blotting as shown in Figure 1. Supernatants from every purification step were analyzed by Western blot and Coomassie Blue staining. All samples from washing steps did not show a band at approximately 29.9 kDa indicating complete binding of this His-tagged recombinant Com1 protein to the Ni-NTA column. Elution fractions 2, 4 and 5 showed clear bands at approximately 29.9 kDa and were combined for size exclusion chromatography. The purified, concentrated recombinant Com1 protein showed a clear band at approximately 29.9 kDa and the protein concentration was determined as 1300 µg/mL. The presence of only a few additional protein bands suggests high purity of the protein preparation used in the following ELISA assays (Figure 1). 

### 3.2. Serum Samples

Sera of 115 sheep from 13 locations, 104 goats from 11 locations and 197 cattle from 16 locations were collected over the last 10 years and tested with commercially available ELISA for Q fever. Overall 60.0% of sheep sera, 17.3% of goat samples and 60.8% of cattle sera were positive. Q fever true positive sera were sampled from 62 sheep sera from four different locations, 11 goat sera from two different locations and 43 cattle sera from three different locations. Sera from 31 sheep from six different apparently healthy flocks, 68 goat sera from six different apparently healthy herds and 27 cattle sera from two different apparently healthy herds showed characteristics for Q fever negative sera.

In total 71 of all 416 sera were taken over with positive results for other bacteria from the respective NRL (23 of 203 Q fever positive sera and 48 of 207 Q fever negative sera). 

Description of all sera is summarized in Table 2 and supplementary Table S1.

### 3.3. Recombinant Com1-ELISA Tested with Field Sera from Sheep

A total of 115 sheep serum samples were tested for reactivity with recombinant Com1 protein in an ELISA (recombinant Com1-ELISA). Four of them were excluded from this study: two sera with suspicious results using commercial ELISA, one serum determined as negative using commercial ELISA but with the highest optical density (OD_450_) value using the recombinant Com1-ELISA and one serum determined as positive with commercial ELISA but having lowest OD_450_ value in recombinant Com1-ELISA. Of the remaining 111 serum samples, 47 were tested negative for *C. burnetii*-specific antibodies and 64 sera were tested positive for *C. burnetii*-specific antibodies by commercial ELISA. The latter sera were collected during confirmed outbreaks of Q fever. For all sera OD_450_ values between 0.099 and 2.250 were obtained using the recombinant Com1-ELISA. As control sera, one positive and one negative field serum of sheep were selected, which showed very high (positive) and very low (negative) OD_450_ values in both the commercial ELISA and the recombinant Com1-ELISA.

**Figure 2:**
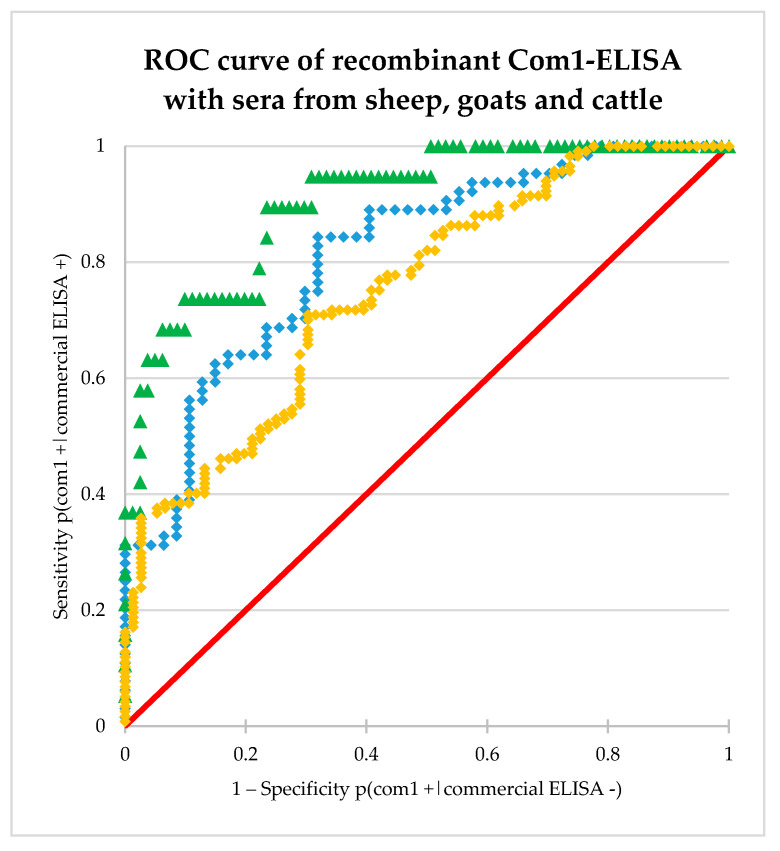
Plot of sensitivity versus 1–specificity for all possible cut-off values. Dots show OD_450_ values of ovine field sera (blue dots), caprine field sera (green triangles) and bovine field sera (yellow squares). Red line shows random distribution in contrast.

Based on the recombinant Com1-ELISA values, a receiver operating characteristic curve (ROC curve) was developed as shown in Figure 2. The integral, the area under the curve (AUC) was 0.82, which means an “excellent discrimination” [59].

The cut-off value was defined at the point with best sensitivity at best specificity and set at 0.32. Of the 111 sheep sera, 54 sera were identified as true positive and 32 sera were found to be true negative. False positive results were shown for 15 sera and false negative results for 10 sera, resulting in a sensitivity of 84% and a specificity of 68% as shown in Table 3.

The 15 sera with false positive results in the recombinant Com1-ELISA were from six geographically different located flocks (number of location as in Table S1; false positive sera/total samples per location: (1) 1/29; (2) 1/4; (4) 2/10; (6) 1/16; (10) 9/10; (11) 1/1). In a previously performed phase I/phase II antibody-specific ELISA (Euroimmun AG) all false positive samples (*n* = 13) of the first four locations named (1); (4); (6) und (10) were tested. Results are indicated as phase-specific positive Ph^+^, negative Ph^−^ or questionable Ph^?^. One of these 13 false positive sera, which were tested against phase-specific antibodies, showed a positive result for phase I antibodies but was negative for phase II antibodies (location (1)) PhI^+^/PhII^−^. Another false positive sample showed a positive result for phase II antibodies but was negative for phase I antibodies (location (6)) PhI^−^/PhII^+^, confirming the recombinant Com1-ELISA results. The former serum was from a defined outbreak, where only two samples showed negative results in the commercial ELISA. The latter serum was from another defined outbreak and the only one having a negative result in the commercial ELISA from this location. All other false positive sera showed negative results for phase I and phase II antibodies PhI^−^/PhII^−^ confirming the commercial ELISA results.

Interestingly one of the false positive sera (location (11)) was a commercially available lamb serum.

The 10 sera with false negative results in the recombinant Com1-ELISA were from four geographically different located flocks (false negative sera/total samples per location: (1) 1/29; (5) 2/10; (6) 5/16; (8) 2/10). In a previously performed phase I/phase II antibody-specific ELISA, three sera (one sample of each location (5); (6) and (8)) were determined as questionable for phase II^?^, but positive for phase I antibodies PhI^+^/PhII^?^. One serum from location (6) was uncertain for phase II antibodies, but negative for phase I antibodies PhI^−^/PhII^?^. All six other tested sera were phase I and phase II positive PhI^+^/PhII^+^ confirming the commercial ELISA results.

The phase I/phase II antibody-specific ELISA confirmed the results of the commercial ELISA in 49% of the cases for positive results and in 26% tests for negative results. In 62% of the positive samples and in 31% of the negative field sera at least one of the tests for phase I or II confirmed the results of the commercial ELISA.

In order to optimize the performance of this ELISA for diagnostics, the cut-off OD_450_ value for negative results was set at 0.21, for positive results at 0.50 and for suspicious at >0.21 and <0.50 leading to a sensitivity and specificity of 89%, respectively.

To test if the recombinant Com1-ELISA is robust against antibodies of other bacteria, the statistical values AUC, specificity and sensitivity were calculated excluding sera positive for *Chlamydia* spp. (*n* = 106). AUC and specificity decreased slightly (AUC: −0.007, specificity: −0.84 percentage point), while sensitivity remained unchanged at the constant cut-off value. With regard to the false positive rate of the sera tested positive for *Chlamydia* spp., it was shown that these sera have similar statistical values (specificity only for *Chlamydia*-positive sera: 75%). This shows that recombinant Com1-ELISA is robust against influences of antibodies against other bacteria like *Chlamydia* spp.

These data imply that a majority (84%) of Q fever positive sheep sera reacted with *C. burnetii* Com1. 

### 3.4. Recombinant Com1-ELISA Tested with Field Sera from Goats

A total of 104 goat serum samples were tested with recombinant Com1-ELISA. Four of them were excluded: two showing suspicious results in commercial ELISA, one was tested negative for Q fever using commercial ELISA, but showing highest optical density (OD_450_) value using recombinant Com1-ELISA and one serum was tested positive for Q fever using commercial ELISA, but having the lowest OD_450_ value using recombinant Com1-ELISA. Of the remaining 100 serum samples, 81 were tested negative for Q fever. The remaining 19 sera were tested positive for Q fever by commercial ELISA. The recombinant Com1-ELISA showed OD_450_ values between 0.061 and 2.090. As control sera one positive and one negative field serum of goats were selected, which showed very high (positive) and very low (negative) OD_450_ values in both, the commercial ELISA and the recombinant Com1-ELISA.

Based on the recombinant Com1-ELISA values, a ROC curve was developed as shown in Figure 2. The integral, the area under the curve (AUC) was 0.91, which means an “outstanding discrimination” [59].

The cut-off value was defined at the point with best sensitivity at best specificity and set at 0.22. Of the 100 goat sera, 17 sera were identified as true positive and 62 true negative using the recombinant Com1-ELISA. Two sera showed false negative and 19 sera false positive results resulting in a sensitivity of 90% and a specificity of 76% as summarized in Table 4.

Two sera with false negative results in the recombinant Com1-ELISA were from two different herds with every serum tested as true positive (false negative sera/total samples from location: (6) 1/6 and (21) 1/9). In a previously performed phase I/phase II antibody-specific ELISA, the sample from location (21) showed a positive result for phase I and a negative result for phase II antibodies PhI^+^/PhII^−^. The sample from location (6) showed an uncertain result for phase I and a positive result for phase II antibodies PhI^?^/PhII^+^.

The 19 sera with false positive results using the recombinant Com1-ELISA were from seven different origins (false positive sera/total samples from location: (12) 1/3; (13) 1/8; (14) 8/32; (15) 1/1; (18) 2/10; (19) 1/10; (20) 5/10). In a previously performed phase I/phase II antibody-specific ELISA, all sera from location (19) and (20) were tested. All seven sera were negative for phase I and phase II antibodies confirming the commercial ELISA results PhI^−^/PhII^−^.

The phase I/phase II antibody-specific ELISA confirmed the results of the commercial ELISA in 27% of the cases for positive and in 55% tests for negative results. In 41% of the positive samples and in 100 % of the negative field sera at least one of the tests for phase I or II showed the respective result confirming the results of the commercial ELISA. 

Nearly 90% of false-positive samples were collected during routine Q fever diagnostic testing (17 samples). Of 11 sera positive for other pathogens, two showed false-positive results with the recombinant Com1-ELISA. 

In order to optimize the performance of this ELISA for diagnostics, the cut-off OD_450_ value for negative results was set at 0.20, for positive results to 0.30 and >0.20 and <0.30 for suspicious results leading to a sensitivity and specificity of 92% and 89% for the recombinant Com1-ELISA with goat sera, respectively.

To test if the recombinant Com1-ELISA is robust against antibodies of other bacteria, the statistical values AUC, specificity and sensitivity were calculated excluding sera positive for other pathogens (*n* = 57). AUC and specificity decreased slightly (AUC: −0.003, specificity: −0.23 percentage point), while sensitivity remained constant at the constant cut-off value. This suggests that recombinant Com1-ELISA is robust against influences of antibodies of other bacteria like *M. avium* ssp. *paratuberculosis* or *Brucella* spp.

These data suggest that the majority (90%) of Q fever positive goat serum samples reacted to *C. burnetii* Com1.

### 3.5. Recombinant Com1-ELISA Tested with Field Sera from Cattle

A total of 197 cattle serum samples were tested with the recombinant Com1-ELISA. Four of them were excluded: two with suspicious results in commercial ELISA, one sample determined as negative using commercial ELISA but with the highest optical density (OD_450_) value using recombinant Com1-ELISA as well as one positive serum in commercial ELISA having lowest OD_450_ value using recombinant Com1-ELISA. Of the remaining 193 serum samples, 76 were tested negative for Q fever and 117 sera were tested positive for Q fever by commercial ELISA. Recombinant Com1-ELISA showed OD_450_ values between 0.07 and 0.57. As control sera, one positive and one negative field serum from cattle were selected, which showed very high (positive) and very low (negative) OD_450_ values in both, the commercial ELISA and the recombinant Com1-ELISA.

Based on the recombinant Com1-ELISA values, a ROC curve was developed as shown in Figure 2. The AUC was 0.75, which means an “acceptable discrimination” [59].

The threshold value was defined at the point with best sensitivity at best specificity and set at 0.18. Of the 193 cattle sera, 83 were found true positive and 52 were identified to be true negative. For 24 sera false positive and for 34 sera false negative results were obtained leading to a sensitivity of 71% and a specificity of 70% as seen in Table 5.

False positive results were shown by 23 sera taken from eight different locations in the recombinant Com1-ELISA (false positive sera/total samples from location: (15) 1/2; (23) 2/9; (22) 3/11; (25) 6/24; (31) 2/5; (32) 2/2; (35) 6/44; (36) 2/14). In a previously performed phase I/phase II antibody-specific ELISA, 17 of the false positive sera were tested (locations: (22), (23), (25) and (36)). All of them were determined as negative for phase I or phase II antibodies PhI^−^/PhII^−^.

False negative results were shown by 34 sera from six different origins in the recombinant Com1-ELISA (false negative sera/total samples from location: (22) 1/11; (24) 9/22; (26) 2/8; (27) 1/11; (34) 15/26; (35) 6/44). In a previously performed phase I/phase II antibody-specific ELISA, 19 of the false negative sera were tested (locations: (22), (27), (26) and (34)). They displayed several different phase-specific patterns such as nine PhI^+^/PhII^+^ (locations: one of (27) and (35), seven of (34)), four PhI^+^/PhII^−^ (one of (22) and three of (34)) and one PhI^+^/PhII^?^ (34). Two false negative sera were from location (34) showing negative results for phase I antibodies and positive PhI^−^/PhII^+^ or uncertain PhI^−^/PhII^?^ results for phase II antibodies, respectively.

The phase I/phase II antibody-specific ELISA confirmed the results of the commercial ELISA in 32% of the cases for positive and in 40% tests for negative results. In 54% of the positive samples and in 100% of the negative field sera at least one of the tests for phase I or II substantiated the results of the commercial ELISA.

In order to optimize the performance of this ELISA for diagnostics, the cut-off OD_450_ value for negative results was set at 0.14 and the minimal cut-off OD_450_ value for positive results to 0.23 leading to a sensitivity and specificity of 91%, respectively. Between these OD_450_ values results are considered as suspicious.

To test if the recombinant Com1-ELISA was robust against antibodies of other bacteria, the statistical values AUC, specificity and sensitivity were calculated excluding sera positive for other pathogens (*n* = 91). AUC, sensitivity and specificity decreased slightly (AUC: −0.04, sensitivity: −6.1 percentage point, specificity: −3.0 percentage point), while the cut-off value remained constant. This suggests that recombinant Com1-ELISA is robust against influences of antibodies against other bacteria like *Chlamydia* spp., *Parachlamydia* spp., *M. avium* ssp. *paratuberculosis* or *Brucella* spp.

The data suggest that the majority (71%) of Q fever positive cattle developed antibodies against *C. burnetii* Com1.

### 3.6. Recombinant Com1-ELISA with Q Fever True Positive Sera

Of the total 404 field sera, 116 sera (62 sheep, 11 goat, 43 cattle sera) were identified to be Q fever true positive.

When calculating sensitivity for these sera with mentioned species-specific cut-offs 51 samples of 62 Q fever true positive sheep sera showed positive results in recombinant Com1-ELISA resulting in a sensitivity of 82.26%. Of the 11 Q fever true positive goat sera, 9 samples were tested positive in recombinant Com1-ELISA resulting in a sensitivity of 81.82%. For cattle sera a sensitivity of 83.72% was calculated with 36 positive results in recombinant Com1-ELISA of 43 Q fever true positive sera. Over all sensitivity of recombinant Com1-ELISA for Q fever true positive sera was 81.51%.

## 4. Discussion

Previous studies have shown that *C. burnetii* Com1 is an immunodominant protein in humans, mice or guinea pigs [48,60]. Antibodies against Com1 are detectable during the early phase of infection especially in mice [48,60]. Surface exposed outer membrane proteins such as Com1 could be promising candidates for new diagnostics. Therefore, the performance of recombinant Com1 used in a prototype ELISA was estimated with Q fever positive and negative field sera from ruminants. 

The results of this study showed that 154 of the 200 tested sera of sheep, goats and cattle, that were previously tested positive with commercial ELISAs, were also positive with the newly developed recombinant Com1-ELISA. Of the 204 negative ruminant sera, 146 samples were also negative with recombinant Com1-ELISA. These results led to an over-all sensitivity and specificity of 77% and 72%, respectively. The low OD_450_ values of some of the positive sera can be explained by the fact that not every animal developed antibodies against *C. burnetii* Com1. In fact, of all 119 Q fever true positive sera 97 were tested positive in recombinant Com1-ELISA resulting in a sensitivity of 82.76%. Inter-species differences were marginal (σ = 0.0081). While commercial ELISAs use whole cell lysates offering a broad variety of antigens, the here developed recombinant ELISA is based on a single, well-defined antigen. However, the vast majority of Q fever true positive animals (83% in total) reacted to recombinant Com1 protein indicating its diagnostic value as already shown for mice and just recently for humans [48,55,61,62,63].

Our data show large differences for the maximum and minimum OD_450_ values obtained from sera of sheep, goats or cattle, respectively. The lowest and highest measured OD_450_ values for sheep sera were 0.099 and 2.225, respectively. This leads to a difference of 2.15. The margins were lower for goat sera (1.49) and lowest for cattle sera (0.49). While an “outstanding discrimination” was achieved for goat sera, sheep sera showed an “excellent discrimination”. With sera from cattle an “acceptable discrimination” was scored. Mathematically, the reasons for this can be found in the large (sheep) or small (cattle) range of OD_450_ values. The results can be graded more precisely the greater the distribution between the OD_450_ values is. Serologically, this could be caused by the individual immune status and immune response of the species or individual animals, respectively, to *C. burnetii* and especially to its surface markers. This leads to the conclusion, that between species and even between individual ruminants different immune reactions to *C. burnetii* Com1 can occur. Peculiarities of different ruminant breeds might be a further factor. But, as the particular breeds of the donors of this study are unknown, this assumption cannot be evaluated.

The sensitivities and specificities calculated here for the recombinant Com1-ELISA with sera from sheep, goats and cattle varied between 71–94% and 68–77%, respectively. These values correspond well with previously published sensitivities and specificities in studies using human sera. Sekeyova et al. showed a sensitivity of 47% and a specificity of 71% for Com1 as antigen using sera from patients suffering from Q fever related endocarditis [63]. Another study calculated sensitivity and specificity for Com1 in an ELISA with human sera from patients with acute and chronic Q fever, naïve as well as vaccinated subjects at 50% and 90%, respectively [54]. Recently, Vranakis et al. described an experimental ELISA based on recombinant Com1 with a sensitivity of 92.9% and a specificity of 92.4% for sera obtained from patients suffering of chronic Q fever [55].

When comparing the statistical rates of the recombinant Com1-ELISA developed here with commercial ELISAs, it should be noted that the latter often include a range for questionable results. This range was deliberately omitted for the recombinant Com1-ELISA for better comparison to other studies. If such a range was included for goat sera (OD_450_ values between 0.20 and 0.30), the sensitivity and specificity would rise to 92% and 89%, respectively. For sheep sera cut-off values for negative results at OD_450_ 0.21 and for positive results at OD_450_ 0.50 would result in sensitivity and specificity of 89%. For cattle sera cut-off values at 0.14 for negative results and 0.23 for positive results would lead to a sensitivity and specificity of 91%.

Whole-cell antigens, which are used for commercial ELISAs, bear the risk of cross reactivity. In particular, cross-reactivity against *Chlamydia* spp. has been previously described using immunoblot techniques [40]. The use of single recombinant proteins in ELISA could reduce the risk of cross-reactions. Four sheep sera were obtained as *Chlamydia* spp. positive. Only one of these was tested false positive for *C. burnetii* using the recombinant Com1-ELISA. Of 19 tested *Chlamydia* spp. positive sera from cattle, 10 were tested true negative and 3 true positive with recombinant Com1-ELISA. So far, only one Com1-like protein has been found in *Legionella pneumophila* [64]. However, findings of a previous study suggest that cross-reactions occur only to a small extent [48]. In that study, none of 10 tested human Q fever-negative, legionellosis-positive serum samples reacted in the used Com1 based microarray. Up until now, it is not possible to estimate if this could lead to cross-reactivity because *L. pneumophila* is not a typical pathogen for ruminants but only for humans. 

The need to detect the early phase of a *C. burnetii*-infection in ruminants as well as the identification of so far serologically negative shedders is necessary for control programs and an important aim for the development of new serodiagnostic test systems. Further testing is needed to show whether *C. burnetii* Com1 would be suitable for this purpose. In order to increase performance of the test and to avoid cross-reactivity, a combination with other recombinant immunogenic proteins is a promising option. According to the literature, GroEL and OmpH would be very suitable candidates [48]. Their overall combined potential should be investigated in further experiments. The results of this study show that Com1 can be used for the development of new more sensitive and specific diagnostic tools for Q fever.

## 5. Conclusions

Recombinant Com1 protein reacts with antibodies from Q fever infected ruminants at a satisfactory percentage and, therefore, could be used as basis for more sensitive and specific diagnostic tools in veterinary medicine. Sensitivities and specificities determined were: for sheep (OD_450_ cut-off value of 0.32) 85% and 68%, for goats (OD_450_ cut-off value of 0.23) 94% and 77% and for cattle (OD_450_ cut-off value of 0.18) 71% and 70%, respectively.

## Figures and Tables

**Table 1:** Bacterial strains and plasmids used in this study.

Strain	Genotype, Characteristics		Origin/Source
*Coxiella burnetii*	Nine Mile Phase II RSA 439		strain collection FLI
*Escherichia coli* TOP10	*F– mcrA D(mrr-hsdRMS-mcrBC) f80lacZDM15 DlacX74 recA1 araD139 D(ara-leu)7697 galU galK rpsL (StrR) endA1 nupG*		Invitrogen
*Escherichia coli* BL21(DE3)	*F– ompT hsdSB (rB–mB–) gal dcm l(DE3 [lacI lacUV5-T7 gene 1 ind1 sam7 nin5])*		Invitrogen
**Vector**	**Function**	**Selection marker**	
pCR™8/GW/TOPO™	cloning vector, *spnR*	spectinomycin	Invitrogen
pET300/NT-DEST	expression vector, *bla*	ampicillin	Invitrogen

**Table 2:** Q fever status of sera from sheep, goats and cattle collected during Q fever outbreaks, from apparently healthy herds or supplied by Friedrich–Loeffler Institute, Federal Research Institute for Animal Health (FLI) National Reference Laboratories (NRL).

	Sera from Sheep (Number of Locations)	Sera from Goats (Number of Locations)	Sera from Cattle (Number of Locations)
**Positive for *C. burnetii* in commercial ELISA**	65 (6)	20 (3)	118 (8)
Q fever true positive	62 (4)	11 (2)	43 (3)
Positive for other bacteria (tested for by respective NRL)	0	0	4 (*Chl. psittaci*) (1) 7 (*M. avium* ssp. *paratuberculosis*) (1) 12 (*Parachlamydia* spp.) (1)
**Suspicious for *C. burnetii* in commercial ELISA**	2 (2)	2 (2)	2 (2)
**Negative for *C. burnetii* in commercial ELISA**	48 (10)	82 (9)	77 (13)
From healthy herds	31 (6)	68 (6)	27 (2)
From serological positive herds	4 (2)	2 (1)	17 (2)
Positive for other bacteria (tested for by respective NRL)	4 (*Chl.* spp.) (1)	10 (*M. avium* ssp. *paratuberculosis*) (1) 1 (*Brucella* spp.) (1)	2 (*Chl. pecorum*) (1) 13 (*Chl. psittaci*) (3) 3 (*M. avium* ssp. *paratuberculosis*) (1) 13 (*Parachlamydia* spp.) (3) 2 (*Brucella* spp.) (1)
**Total tested**	115 (13)	104 (11)	197 (16)
Excluded from analysis because of lowest/highest density in recombinant Com1-ELISA	2 (2)	2 (2)	2 (2)
**Total in analysis for immunodominance**	111 (13)	100 (11)	193 (16)

**Table 3:** Statistical values for the recombinant Com1-ELISA at the determined threshold value of 0.32 for sheep sera. The contingency table shows the number of true and false negative as well as true and false positive results for sheep serum samples.

Sheep Sera		Results Commercial ELISA		
Results recombinant Com1-ELISA		Positive	Negative	Total
	Positive	54	15	69
	Negative	10	32	42
	Total	64	47	111

**Table 4:** Statistical values for the recombinant Com1-ELISA at the determined threshold value of 0.22 for caprine sera. The contingency table shows the number of true and false negative as well as true and false positive results for goat serum samples.

Goat Sera		Results with Commercial ELISA		
Results with recombinant Com1-ELISA		Positive	Negative	Total
	Positive	17	19	36
	Negative	2	62	64
	Total	19	81	100

**Table 5:** Statistical values for the recombinant Com1-ELISA at the determined threshold value of 0.18 for cattle sera. The contingency table shows the number of true and false negative as well as true and false positive results for cattle serum samples.

Cattle Sera		Results with Commercial ELISA		
Results with recombinant Com1-ELISA		Positive	Negative	Total
	Positive	83	24	107
	Negative	34	52	86
	Total	117	76	193

## Supplementary Materials

The following are available online at https://www.mdpi.com/2076-2607/8/8/1235/s1.
